# “Psychology’s Misuse of Statistics and Persistent Dismissal of its Critics”

**DOI:** 10.17505/jpor.2025.28098

**Published:** 2025-06-28

**Authors:** Lars-Gunnar Lundh

**Affiliations:** Department of Psychology, Lund University, Sweden

**Keywords:** Variables, Individuals, Individuality, Probability, Psychodemography, Population psychology, Population genetics

James T. Lamiell is professor emeritus in psychology at Georgetown University in Washington DC, USA. In this book he summarizes his critique of mainstream psychological research, which he claims is based on a

deep, abiding, and highly problematic confusion… that statistical knowledge about variables defined only for aggregates of individuals entitles scientifically authoritative claims to knowledge about the individuals within those aggregates. (p. 1)

For simplicity’s sake, I will here refer to this kind of confusion as the “variable/individual confusion”. Similar critiques have been raised from different perspectives by several other writers as, for example, Molenaar (2009), Bergman and Andersson (2010), Richters (2021), Moeller (2021), and van der Gaag (2023). But Lamiell in some respects goes farther than these other critics.

The variable/individual confusion, according to Lamiell, has serious and far-reaching consequences both for psychological science (by producing *bad science*) and for professional psychology (by producing *bad professional practice*). In fact, says Lamiell, much mainstream psychological research does not even deserve to be called *psychological* research, but rather represents a kind of demographic research, which he refers to as “psychodemography”.

These are important things that need to be discussed in some detail. In the present review, I will focus on four aspects of Lamiell’s reasoning: (1) the need to shift focus from variables to individuals; (2) the challenge of studying individuality; and (3) confusions about probability; and (4) the notion of psychodemography. Although I agree with much of Lamiell’s reasoning, he also makes some claims that I disagree with.

## Shifting Focus from Variables to Individuals

Lamiell recounts a crucial experience from early in his career when he was teaching students about the results from correlational studies in personality research. He describes how he told his students about research findings which showed that even the best measures of personality traits correlated only around *r =.30* with behaviours that express these traits. When Lamiell explained to his students that this correlation meant that the personality measures accounted for, at best, merely 9% (*r^2^* = .09) of the between-person variance in the behavioural measures, his students challenged him with questions that would eventually lead to a profound shift in his own way of thinking:

my students would implore me to make clear to them just how a correlation coefficient or its square, ‘percent variance accounted for’, spoke to the question at hand. They would ask, for example, if the finding *r^2^* = .09 meant that 9% of the individuals investigated in some study were consistent and 91% of those investigated were not. Of course, my reply had to be that, no, the *r^2^* statistic could not be interpreted in exactly that way. ‘Well, then’, they would ask, does that statistic indicate that individuals are consistent 9% of the time and inconsistent 91% of the time? ‘Well, no’, I was obliged to respond, ‘the *r^2^* statistic does not mean exactly that either.’ (Lamiell, 2019, p. 25)

Lamiell soon realized that, in fact,

no scientifically valid explanation of the meaning of *r* or *r^2^* could be given that would answer the questions my students were asking me. The statistics in question were defined for *variables* marking *individual differences*, and at the level of the individual there is *no such thing* as an empirically specifiable individual *difference*. (Lamiell, 2019, p. 26)

What this example shows is, first of all, that this kind of research, as presented in terms of correlations and percent variance explained, does not say anything about *individuals*. But does this mean that it is “bad science”? It does say some-thing about the relations between psychological variables. The question then might rather be how *relevant* this kind of knowledge about relations between variables is to the development of psychological science. But, of course, it *is* bad science if empirical findings about the relations between *variables* are interpreted as facts about *individuals*.

The data from much variable-oriented research can, in fact, be reanalyzed by changing the focus from variables to individuals. For example, the questions that were asked by Lamiell’s students can in principle be answered by using a scattergram to visualize how the correlation looks for each individual in the study. This might make it possible to say what percentage of individuals were consistent and what percentage of individuals were not. Lamiell himself, in fact, provides an illustrative example on p. 27-29 in his book of how the correlation between *variables* may hide apparently contradictory facts about the percentage of *individuals* who show a certain pattern of values on variables.

It is quite possible to change the focus from variables to individuals also in experimental research that compares an experimental group with a control group. Lamiell provides a good example of this in Chapter 7, where he refers to Grice’s (2015) model for reanalyzing findings from studies in experimental social psychology, where the hypothesis was originally tested only at the group level by a statistical comparison of the means of independent groups. In Grice’s model, the investigator is required

to make explicit what should be observed of any given participant in order for that participant to be regarded as having confirmed – or not – the hypothesis. The data are then tallied on a case-by-case basis. (Lamiell, 2019, p. 158)

This procedure seems analogous to what is often done in randomized controlled trials (RCTs) in psychotherapy research, when the results for each patient are analyzed in terms of pre-determined criteria for whether the individual is “recovered” or at least shows *clinically significant improvement*.

Unfortunately, Lamiell sometimes writes as if RCTs only involve comparisons of mean change at the group level:

As is true of all treatment group experimentation, the *findings* of RCTs are defined by the outcome of statistical comparisons of treatment group averages. (Lamiell, 2019, p. 158)

This is not quite true. The most interesting findings reported from RCTs are the percentages of patients who recover or show clinically significant improvement. For example, a large meta-analysis of RCTs (Cuijpers et al., 2023) showed that 42% of the patients in cognitive-behaviour therapy (CBT) responded to treatment, while the response rate was only 19% in the control groups. This is a report about individuals, not about relations between variables.

All the examples above help to illustrate how it is possible to shift focus from *variables* to *individuals* even with a given dataset, both in correlational and experimental studies. Although the data are the same, they can be viewed either from a variable-view or an individual-view. The data are at the population level, and the shift that takes place is from a focus on the “*relation between variables in populations of individuals*” to a focus on “*populations of individuals*” (Lundh, 2023, p. 78).

Still, this kind of focus on individuals is of a very limited sort and does not involve any understanding of individual *persons* of the kind Wilhelm Stern (1900) had in mind when he proclaimed *individuality* as the big challenge for psychological science in the 20^th^ century. Lamiell (2012, 2021) has written extensively about Stern’s contributions to psychological science, and the present book provides a brief but informative summary of Stern’s approach, that should be of interest to anyone who is interested in person-level psychology.

## The Challenge of Individuality

Lamiell describes how Stern (1911) differentiated between (1) research on the variation and correlation of psychological attributes *across individuals* (i.e., population-level research) and (2) research on patterns of psychological attributes *within individuals* (i.e., person-level research), while emphasizing that the former can in no way substitute for the latter. Stern spoke of two varieties of person-level research: *psychography* and *comparative research*. Psychography was defined as “the empirical specification of the psychological features of a person’s individuality”, whereas comparative research involves a comparison of two or more individuals in terms of their psychological attributes. Lamiell also points out that, in Stern’s view, quantitative *psychographic* investigations were not the end point but should serve as a preliminary for genuine *biographic* studies of a more qualitative nature.

What became of Stern’s psychography and comparative studies? Lamiell tells us how psychological science took another turn by following Thorndyke’s approach to differential psychology, which effectively obliterated the distinction between persons and variables. According to Thorndyke,

the correlation between two variables measuring between-person differences indicates “the extent to which the amount of one trait possessed *by an individual* is bound up with the amount *he* possesses of some other trait” (Thorndyke, 1911, p. 22, emphasis added). (Lamiell, 2019, p. 59)

Here we see a clearcut illustration of the variable/individual confusion. It is an interesting question how such a confused way of thinking could take hold in scientific research, while Stern’s thinking about these matters fell into oblivion.

How should we, now well into the 21^th^ century, evaluate Wilhelm Stern’s (1900) proclamation of individuality as the big challenge for psychological science in the 20^th^ century? Was he completely wrong? No, it may be argued that individuality *did*, in fact, become a main theme in psychological thinking during the 20^th^ century, but mainly *outside of academia*. Those who were interested in individuality could turn to other varieties of psychological theory, less based on empirical research, such as psychoanalysis and humanistic psychology, which came to flourish during the 20^th^ century.

Although Lamiell does not write about these developments in his book, it seems clear that *clinical* psychologists have an essential and undeniable need to understand the functioning of individual persons. During the 20^th^ century this resulted in the development of a wild flora of psychoanalytic, psychodynamic, humanistic and existential theories about the development and dynamics of individual persons and interpersonal relations. During the second half of the 20^th^ century this was complemented by the development of behaviour therapy and CBT, where the corresponding need for an understanding of individuality was filled by the development of paradigms for individual behaviour analysis, functional analysis, and other varieties of conceptualization of the idiosyncratic patterns of functioning of individual patients, that could serve as the basis for tailor-made treatments of their psychological problems.

The challenge that lies before us is probably to develop *a scientific basis for the study of individuality*, including both individual development and interpersonal relations. Some leads for such development probably may be found in Wilhelm Stern’s work, whereas other leads may be found in phenomenology, theories of dynamic systems, and various sub-areas of mainstream psychological science. My guess is that the main task here is to develop an adequate conceptual framework for the understanding of individuality, individual development and interpersonal relations, that can provide a unified paradigm for person-oriented research.

Lamiell’s focus in this book, however, is more on statistics than on psychological theory. In particular, he focuses on the concept of probability, and a certain kind of confusion in this area which, he says, plague professional psychology and leads to *bad professional practice*.

## Confusions About Probability

A relatively large part of Lamiell’s book is devoted to confusions about probability. In particular, Lamiell addresses the assumption that “statistical studies of variables defined for populations can warrant *probabilistic* knowledge claims about the individuals within these populations” (Lamiell, 2019, p. 111). For example, he argues that although doctors may be well-advised to prescribe penicillin to their patients, based on data that about 85% of the patients tend to improve on this treatment, this is a finding on the *population* level, that does “not justify any claim by the doctor to know that that the patient standing in front of her today ‘has an 85 percent chance’ of improving (p. 117). The claim of an ‘85% chance’ simply *does not apply* at the level of the individual:

probabilistic knowledge is always and of its very essence factual knowledge about a *series* of instances considered as a single entity. It cannot properly be regarded as knowledge about any single instance within that series. (Lamiell, 2019, p. 130).

Moreover, Lamiell contends that such interpretations of probabilistic findings lead to *socio-ethical problems*, when results from randomized controlled studies (RCTs) lead to public policies that prescribe specific treatments for patients with specific kinds of problems. According to Lamiell, the fact that a treatment shows significant effects on the *population* level should not be taken as an argument for how specific *individuals* should be treated. Here, however, it becomes difficult for me to follow Lamiell’s reasoning. On the same logic, he should also claim that any public policies for prescribing evidence-based *medical* treatments are socio-ethically problematic even when the data show an improvement rate of 85% (as in the penicillin example above).

There are many problems with RCTs in psychotherapy research, but Lamiell seems to miss the most pressing problems. As summarized elsewhere, *unlike medical treatments*,

the treatments studied in psychotherapy research represent large *treatment packages* involving many different interventions and interactions over a considerable time period… The therapies that are tested in RCTs are not described in terms of observable treatments (as is common in other areas of experimental psychology) but in terms of certain constructs that are used to label entire treatment packages (e.g., “cognitive behavior therapy”, “short-term psychodynamic therapy”, “interpersonal therapy”, etc.), the principles and procedures of which are outlined in manuals. (Lundh, 2023, p. 83)

This means that RCTs suffer from a low degree of control of the experimental “manipulation”, to such an extent that the design may even be questioned as to whether it deserves to be labelled “experimental” in the strict sense. This also makes it impossible to draw theoretical conclusions about what is at work in these treatments.

The probability issue may be a lesser problem. To return to the example of RCTs in research on the treatment of depression that was briefly described above: In a large meta-analysis, Cuijpers et al. (2023) studied the effects of CBT for depression by including 409 RCTs (518 comparisons) with 52,702 patients. Among other things, the findings showed (1) that CBT was more effective than control conditions such as usual clinical care and waitlist conditions. In terms of percentages, 42% of the patients in CBT responded to treatment, while the response rate was only 19% in the control groups. (2) In the short term, the effects of CBT were comparable to those of pharmacotherapies, but at 6-12 months follow-up CBT was significantly more effective. (3) The comparison to other psychotherapies showed a very small differential effect, which was not sufficiently robust to remain significant in sensitivity analyses. On the *population* level, this seems to be grounds for making some recommendations about the treatment of depressed patients (e.g., that psychotherapy is better than no treatment, and in the long term probably more efficient than pharmacotherapy, although there is little evidence that any one form of psychotherapy is better than another), whereas at the level of the *individual* it is impossible to know if a given patient will benefit from the recommended treatment.

Lamiell is obviously quite correct when he argues that probabilistic findings at the population level cannot be *generalized* to the individual case. Yet, it is difficult to agree with him when he seems to conclude that evidence at the population level *has no bearing at all* on the individual case.

These issues have been extensively discussed by other researchers and philosophers, and it would have been interesting if Lamiell had included some arguments from their discussion.

Bayesian thinkers such as David Lewis have argued, in contrast to Lamiell, for what they refer to as the Principal Principle, according to which our credence in a single case *follows* from the general probability of all such cases. Others have argued against this principle, but without drawing the same conclusions as Lamiell. In a sharp and thoughtful paper, for example, James (2010) has argued that even if data on probability derived from population-level research does not *apply* to the single case, these data can still *guide* the individual. Both camps agree that it is a wise thing to follow treatment guidelines based on population-level data.

In contrast, Lamiell, argues against such a conclusion. He even argues – and this seems to be his most controversial claim in the book – that such treatment recommendations involve socio-ethical problems and should be *resisted*:

If practicing clinicians – whatever their level of experience – are inclined to resist such treatment prescriptions, this is just as it should be, and any movement on the part of mainstream psychologists to oppose the resisting clinicians in their efforts would be not only epistemologically unjustified but, as well, socio-ethically questionable. (Lamiell, 2019, p. 132)

To return again to the findings from RCT studies on the treatment of depression: Current treatment prescriptions based on this research involve recommendations of “behavioral therapy, cognitive therapy, cognitive-behavioral therapy (CBT), interpersonal psychotherapy (IPT), mindfulness-based cognitive therapy, psychodynamic therapy, or supportive therapy” (American Psychological Association, 2021). If Lamiell is to be taken literally, when he argues that “if practicing clinicians – whatever their level of experience – are inclined to resist such treatment prescriptions, this is just as it should be” (p. 132), the implication seems to be that *anything* goes, including treatments for which there is no evidence from population-level research, such as for example, rebirthing therapy, past lives therapy, exorcism, and homeopathy. Perhaps this can be seen as a *reductio ad absurdum* of his reasoning in this area.

## Psychodemography or Population Psychology?

Another of Lamiell’s drastic claims is that research on the population level doesn’t even deserve to be labelled as *psychology*. In his view, population-level research on psychological phenomena rather represents a form of demography: *psychodemography*.

Given that the knowledge that mainstream psychological researchers *actually* (as opposed to allegedly) produce by means of their currently favored statistical exercises is knowledge of populations and not knowledge of individuals within those populations, it is fair to say that the field that was once psychology has been transformed into a kind of *psychodemography*. It is a discipline that is *nominally* psychological in that the variables defined for investigation reflect a theoretical interest on the part of investigators in the psychological doings of individuals. Nevertheless, the discipline is *essentially* demographic because its paradigmatic statistical methods are suited only to the production of knowledge about populations. (Lamiell, 2019, p. 18)

The *organizational* implications of this view would be that most work that is done in departments of psychology at present had better be transferred to departments of demography, whereas future research in departments of psychology should only be focused on individuals. Of more immediate interest here, however, are the *theoretical* implications of this view.

In previous papers (Lundh, 2023, 2024), I have argued against Lamiell’s view, by making an analogy between psychology and genetics: Similar to the common division of genetics into three subdisciplines (where *population genetics* is one; e.g., Pierce, 2020). I suggested that psychological science may be seen as having three branches: person psychology, population psychology, and mechanism psychology. That is, just as population genetics is an important subdiscipline of genetics, we may see *population psychology* as an important subdiscipline of psychology. Lamiell, however, maintains that *all* kinds of population-level research, not only in psychology but also in genetics, belong to the science of *demography*. As he puts it,

Strictly speaking, the knowledge provided by “population genetics” is not knowledge of genetics, which is a sub-specialty of biology, but is, rather, knowledge defined by some statistical feature of a genetically determined phenomenon within a population. (Lamiell, 2024b, p. 103)

This is very much at odds with what researchers within genetics say about their science. Population genetics developed in the 1920s and 1930s, as an integration between the principles of Mendelian genetics and Darwin’s theory of natural selection. This, which is often referred to as the “Modern Synthesis” in biology, actually puts population genetics *at the very center of biological science:*

Population genetics is intimately bound up with the study of evolution and natural selection, and is often regarded as the theoretical cornerstone of evolutionary biology. This is because “evolution” has traditionally been defined as any change in a population’s genetic composition…

For a population to evolve by natural selection, the members of the population must vary—if all organisms are identical, no selection can occur. So for selection to gradually modify a population over a long period of time, in the manner suggested by Darwin, a continual supply of variation is needed. (Okasha, 2024)

If population genetics is *the theoretical cornerstone of evolutionary biology*, it can hardly be relegated to a section of demography, as Lamiell would have it.

To pursue the analogy between psychology and genetics even further, in a more speculative direction, the following question deserves to be asked: Could it be that population psychology carries the promise of becoming a discipline at the very center of *social* science, just as population genetics is at the center of *biological* science? Cultures and societies are made of individuals, and individuals show *variation* in their values, attitudes, beliefs, and other psychological characteristics. Moreover, the values, attitudes and beliefs of individuals *change over time*, and they also *differ* between different sociocultural regions.

Consider, for example, the research done with the World Values Survey (e.g., Inglehart, 2021) on the prevalence and change over time of (1) traditional-religious values versus secular-rational values and (2) profertility values versus self-expression values, in different parts of the world. The predominance of different kinds of individual values in different sociocultural regions may be related in interesting ways to the development of different kinds of social institutions such as family, government, religion, education, and media.

To take another example, Twenge and associates (e.g., Twenge et al., 2012) have compared generations of American college students from the 1960s well into the 21^th^ century and have documented several changes in their psychological characteristics. For example, they have found generational increases in self-esteem, assertiveness, narcissism, high expectations, and agency, whereas they report that attributes such as empathy, cooperativeness, and spirituality, have either decreased or remain unchanged. They summarize their results as

consistent with other research revealing generational increases in individualistic traits and agentic self-views …and accounts noting a cultural shift in the United States toward greater individualism (Twenge et al., 2012, p. 420).

How should these changes be explained, and what is their significance for social change? To take yet another example: psychological research suggests that levels of perfectionism have increased over the last decades, so that

recent generations of young people perceive that others are more demanding of them, are more demanding of others, and are more demanding of themselves (Curran & Hill, 2019, p. 410).

How should this be explained, and what are the consequences? Curran and Hill (2019) suggest that the rising experiences of perfectionistic demands may be related to the increasing levels of depression, anxiety, suicide, lone-liness, eating disorders, and body dysmorphia among young people that has been reported recently.

In our own research (Daukantaité et al., 2025), we have seen considerable increases over the past two decades in the prevalence (approximately a doubling) of both non-suicidal self-injury and disordered eating among young girls. How should this be explained? And what are the social consequences?

More examples could be given of time trends at the population level. Most of these findings, however, suffer from a focus merely on *variables*, and not on individual persons seen from a more holistic view. What would be hoped for next is research with a more person-focused approach to explore how all these changes *combine in patterns at the level of the individual*.

Of essential importance here are also theoretical development that can make us synthesize population-level research in psychology with research in sociology, political science, economics and other social sciences. One attempt in such a direction is Markus and Kitayama’s (2010) *mutual constitution model of culture and the self*, according to which

cultures and selves define and build upon each other in an ongoing cycle of mutual constitution (Markus & Kitayama, 2010, p. 420).

Much work in this area, however, remains until (if ever) population psychology will obtain a place at the center of social science.

## Is Psychology Only About Individuals?

When reading Lamiell’s book I often have the impression that he seems to take for granted that psychology *can only be about individuals*, never about populations of individuals. Yet, he speaks positively about the nomothetic search for *general laws* which apply to all people, as for example when he describes Wundt’s original form of experimental psychology. This is confusing to me, because Lamiell does not seem to think that the study of *individual variation* (individual differences) belongs to psychology. Why should it be psychology only when we identify psychological characteristics that apply to *all* people, but not when we study psychological characteristics where there is individual *variation*?

I fully agree with Lamiell when he emphasizes the difference between *general laws* and *statistical generalizations*. He is not the first researcher to have voiced concerns about obliterating this distinction. As he points out, David Bakan raised similar concerns in the 1950s when he claimed that we must differentiate between *general-type* statements and *aggregate-type* statements, and that the failure to do so “is at the root of a considerable amount of confusion which currently prevails in psychology” (Bakan, 1955, p. 211). But it has not been easy to find general laws that apply to all individuals. It is a bit surprising that Lamiell does not discuss *individual variation*, and the possibility of identifying *sub-groups* of individuals who show similar patterns of experiencing and acting, more than he does.

To summarize: This is a book where the author formulates many important questions about the nature and development of psychological science. It is a delight to read certain parts of the book. Still, the book leaves me with mixed feelings. Although I agree with much of Lamiell’s critique of present-day psychological science, he also makes claims that I don’t agree with. And, although his book raises highly important questions about the *history* of psychology, it has little to say about possible avenues for psychological science in the *future*. Although much of Lamiell’s criticism of mainstream psychological research deserves detailed discussion, I would also have liked to see more discussion of how we should proceed into the future.

*Lars-Gunnar Lundh*

## Open access

This article is distributed under the terms of the Creative Commons Attribution 4.0 International License (http://creativecommons.org/licenses/by/4.0/), which permits unrestricted use, distribution, and reproduction in any medium, provided you give appropriate credit to the original author(s) and the source, provide a link to the Creative Commons license, and indicate if changes were made.
